# Management of Chronic Disease and Hospitalization Due to Diabetes among Type 2 Diabetes Patients in Korea: Using the National Sample Cohort Data 2002–2013

**DOI:** 10.3390/ijerph15112541

**Published:** 2018-11-13

**Authors:** Sang Ah Lee, Woorim Kim, Sarah Soyeon Oh, Jieun Yang, Jieun Jang, Eun-Cheol Park

**Affiliations:** 1 Department of Public Health, Graduate School, Yonsei University, Seoul 03722, Korea; ivory0817@yuhs.ac (S.A.L.); wklaura@gmail.com (W.K.); Sarahoh@yuhs.ac (S.S.O.); yje3210@yuhs.ac (J.Y.); jieun99@yuhs.ac (J.J.); 2 Institute of Health Services Research, Yonsei University, Seoul 03722, Korea; 3 Department of Preventive Medicine, Yonsei University College of Medicine, 50 Yonsei-ro, Seodaemun-gu, Seoul 03722, Korea

**Keywords:** management of chronic disease, diabetes, self-management, chronic disease, hospitalizations

## Abstract

To prevent negative outcomes for diabetes patients, developing self-management skills is imperative. This study aimed to examine the association between management of chronic disease (MCD), which mainly involves educating patients about their chronic diseases for obtaining self-management skills and hospitalization due to diabetes among type 2 diabetes patients in Korea. Korean National Health Insurance Service National Sample Cohort data from 2002 to 2013 were used. A total of 54,031 type 2 diabetes patients were included in the study. If patients received the MCD within 1 year from the onset of diabetes, we categorized them as “MCD received patients” We reclassified these groups into five groups: “non-receiving”, “1–3 times”, “4–6 times”, “7–9 times” and “10–12 times” The dependent variable of this study was hospitalization due to diabetes. Cox proportional hazard regression was used. Of the patients, 86.2% (n = 46,571) did not received the MCD within the 1 year from the onset of diabetes. The number of MCDs received increased and the hazard ratio (HR) for hospitalization due to diabetes decreased; particularly, patients who received MCD 10–12 times per annum showed the lowest HR for hospitalization due to diabetes compared to patients in the MCD non-received group (1–3 times per annum: HR: 0.81, *p* = 0.0001; 4–6 times per annum: HR: 0.82, *p* = 0.0248; 7–9 times per annum: HR: 0.75, *p* = 0.0054; 10–12 times per annum: HR: 0.61, *p* < 0.0001). Considering the importance of raising self-managing diabetes skills, the findings can aid in determining the outcomes of the MCD program.

## 1. Introduction

In recent decades, diabetes has become a major health issue with an increasing trend worldwide [1]. It is projected that 592 million people will have diabetes by 2035 [2]. A similar trend is observed in South Korea [3]. In 2011, approximately 4 million adults aged over 30 years had diabetes, which accounted for 1 in 8 adults in South Korea [3]. Including those who are at higher risk of developing diabetes due to impaired fasting glucose, this number has increased to almost 1 in 3 adults [3].

With the increasing prevalence of diabetes, a range of adverse effects has also emerged including the onset of complications, hospitalizations and mortality [4], which can inevitably increase the disease burden [5]. In practice, the cost of care for diabetes patients is estimated to be 1245 million dollars while the cost of inpatient care for diabetes patients is estimated to be 197 million dollars in 2014, with increasing trends in South Korea [6]. Especially, the hospitalization rate of diabetes patients was 37.6 individuals per 100,000 individuals in 2005. It increased to 67.3 individuals in 2013, which was twice the average of the Organization for Economic Co-operation and Development countries [7]. Therefore, negative outcomes including hospitalizations must be prevented in diabetes patients.

Diabetes self-management education has been considered an important part of diabetes management [8]. Because routine care in diabetes is usually handled by patients themselves [9], it is important to know how to accurately self-manage and education regarding self-management is needed. By educating diabetes patients, several positive outcomes could be derived including improving healthy lifestyle behaviors such as smoking cessation [10], improving dietary intake and engaging in physical activities [11]. In addition, education could enhance diabetes patients’ medical or treatment compliance [12], preventing the onset of diabetes complications [13]. Thus, self-managing their disease could prevent disease progression.

In practice, educating diabetes patients to self-manage their disease has been recognized globally as one of the important factors for type 2 diabetes management [14,15,16]. In South Korea, the government has been calculating the “management of chronic disease (MCD)” fee since 2002 in an attempt to encourage primary care physicians to educate patients to understand the course of their disease and, ultimately, self-manage their disease [17]. MCD institutionalizes an ancillary way by which health professionals could educate chronic disease patients to improve their health behaviors. Such behaviors include regular intake of medication, cessation of smoking and drinking, engagement in appropriate physical activity and consumption of recommended dietary intake. Physicians then proceed to write the details of the patient education in their medical charts. The claims are then sent to the Health Insurance Review & Assessment (HIRA), which reviews the claims and the health facility can receive the MCD fee by certifying this medical chart. Therefore, MCD could contribute to the enhancement of self-management skills among diabetes patients.

Although MCD is one of the methods for improving the self-management behavior of chronic disease patients, the effect of this method is not well established thus far. Considering the increasing hospitalization rate of diabetes, there is a need to investigate the association between MCD incentive program and hospitalization. Therefore, this study aimed to examine the association between MCD and hospitalization due to diabetes among diabetes patients in South Korea. Our study hypothesized that diabetes patients who do not have record of MCD are more likely to experience hospitalization due to diabetes.

## 2. Methods

### 2.1. Data and Study Population

We used the Korean National Health Insurance Service National Sample Cohort (NHIS-NSC) data from 2002 to 2013. The NHIS-NSC data includes all medical claims from 1,025,340 individuals, which accounts for 2% of the South Korean population by random sampling. Our study population included those who visited a medical institute that manages type 2 diabetes patients (International Classification of Diseases (ICD) codes: E11) (n = 126,738). Among them. we excluded the patients who had diabetes in 2002, during the washout period, to identify individuals with newly diagnosed diabetes and whose diabetes onset year is 2013 for obtaining at least 1 year of follow-up (n = 31,989). Then, we also excluded those with diabetic complication codes before the onset of diabetes (n = 40,718). Hence, a total of 54,031 diabetes patients were included in the final study. NHIS-NSC data is secondary data and does not contain any data which can identify individual. Therefore, ethical approval is exempted. The requirement for informed consent was waived because the study was based on routinely collected administrative or claims data.

### 2.2. Variables

#### Dependent Variable

The dependent variable of this study was hospitalization due to diabetes among type 2 diabetes patients. Since we followed-up 1 year from the onset of diabetes to determine MCD reception, patients who received inpatient care due to diabetes after the first year of onset of diabetes were categorized as having “experienced hospitalization”. Patients with no record of inpatient visit were categorized as “did not experience hospitalization”.

### 2.3. Calculating Management of Chronic Disease Fee

Calculating MCD fee aims to encourage physicians to educate patients to self-manage their disease to prevent disease progression. It is calculated when physicians at local clinics educate chronic disease patients about their disease and help them develop self-management skills. The target disease group includes the following chronic diseases: hypertension (ICD: I10–I13, I15), diabetes (E10–E14), mental or behavior disorder (F00–F99, G40–G41), respiratory tuberculosis (A15–A16, A19), heart disease (I05–I09, I20–I27, I30–I52), cerebrovascular disease (I60–I69), nervous system disease (G00–G37, G43–G83), malignant cancer (C00–C97, D00–D09), thyroid disorder (E00–E07), hepatic disease (B18–19, K70–K77) and chronic renal disease (N18). The MCD fee can be calculated 12 times a year (two times a month) for one patient per local clinic. Physicians document the details of the patient education in the medical chart while educating the chronic disease patients. When claiming the medical record, they submit the medical chart to prove that they conducted the patient education. After the government organization, HIRA, which reviews the claims, acknowledges the receipt of the chart, the medical facility to which the physician belongs will receive the MCD fee [17]. In the analysis, patients were classified as “MCD received group” if the data on the patients’ medical history contained “AH200” codes with ICD ‘E11’ code within 1 year of diabetes onset. If no “AH200” codes were provided within 1 year from diabetes onset, we classified patients as “MCD non-received group”. We reclassified these groups into five groups: “non-receiving”, “1–3 times”, “4–6 times”, “7–9 times” and “10–12 times”.

### 2.4. Covariates

Age, gender, income status, residential area, insurance status, existence of disability, existence of diabetic complication, Charlson comorbidity Index and year of diabetes onset were used as covariates in this study. Patients’ age was classified into four groups: ≤49 years, 50–59 years, 60–69 years and ≥70 years. Income status was classified into three groups based on health insurance premiums: bottom 20% of premiums, “low-income group”; 20% to 80%, “middle-income group”; and top 20%, “high-income group”. Patients’ residential area was classified into “capital area”, “metropolitan area”, and “rural area”. If patients developed complications after the onset of diabetes, they were categorized as “having complications”. We used the Charlson comorbidity index to identify patients’ comorbidities [18].

### 2.5. Statistical Analyses

To examine the distribution of each variable, we used a chi-square test to examine the frequencies and percentages. To examine the association between receiving MCD and hospitalization due to diabetes, we used the Cox proportional hazards model, adjusted for several confounders. We conducted the Schoenfeld residuals test to check the proportional hazards assumption and found that this assumption was not violated. All analyses were performed using SAS software (ver. 9.4; SAS Inc., Cary, NC, USA).

## 3. Results

The general characteristics of the study population are shown in Table 1. Of 54,031 diabetes patients, 12.8% (n = 6891) experienced all-cause hospitalization. With regard to MCD, 6.8% (n = 3694) of patients received the MCD 1–3 times, 2.4% (n = 1284) of them received the MCD 4–6 times, 2.2% (n = 1164) of them received the MCD 7–9 times and 2.4% (n = 1318) received the MCD 10–12 times within 1 year from the onset of diabetes whereas 86.2% (n = 46,571) did not receive the MCD. The general characteristics of the study population by MCD recipient are shown in Table S1.

Table 2 shows the factors associated with hospitalization due to diabetes using the Cox proportional hazards regression model. The number of MCDs received increased and the hazard ratio (HR) for hospitalization due to diabetes decreased; particularly, patients who received MCD 10–12 times per annum showed the lowest HR for hospitalization due to diabetes compared to patients in the MCD non-received group (1–3 times per annum: HR: 0.81, *p* = 0.0001; 4–6 times per annum: HR: 0.82, *p* = 0.0248; 7–9 times per annum: HR: 0.75, *p* = 0.0054; 10–12 times per annum: HR: 0.61, *p* < 0.0001). As other factors tend to increase, such as age, the hazard ratio for hospitalization due to diabetes also increased (50‒59 years old: HR: 1.48, *p* < 0.0001; 60‒69 years old: HR: 2.03, *p* < 0.0001; ≥70 years old: HR: 3.29, *p* < 0.0001). Compared with those who live in the capital area, those who live in the metropolitan area (HR: 1.22, *p* < 0.0001) or the rural area (HR: 1.41, *p* < 0.0001) were more likely to experience hospitalization due to diabetes. The association between the MCD and all-cause hospitalization is shown in Table S2.

The results of the subgroup analyses of the association between MCD and hospitalization due to diabetes are shown in Table 3. When stratifying by income, the low-income group showed the lowest HR for hospitalization due to diabetes as they received the MCD 10–12 times per annum (HR: 0.46, *p* = 0.0010) compared with the high-income (HR: 0.69, *p* = 0.0399) or middle-income group (HR: 0.68, *p* = 0.0179). With regard to residential area, those who live in the capital area and received the MCD 10-12 times per annum showed a lower HR for hospitalization due to diabetes (HR: 0.58, *p* = 0.0029) than those who live in the metropolitan area (HR: 0.63, *p* = 0.0187) or rural area (HR: 0.64, *p* = 0.0136).

## 4. Discussion

In this study, we examined the association between MCD, which involves self-management education for chronic disease and hospitalization due to diabetes among type 2 diabetes patients in South Korea. We found that patients who received the MCD were less likely to experience hospitalization due to diabetes than those who did not receive the MCD within the first year of diabetes onset. According to the subgroup analyses stratified by age, income, gender, residential area and presence of complications, this association was even higher for the low-income group. These results indicate that MCD is positively associated with hospitalization due to diabetes by enhancing patients’ self-management of diabetes.

In chronic disease patients, self-management is considered inescapable [19]. Especially, self-management is inevitable for diabetes patients to control their blood glucose. Previous studies showed that improving self-management skills could avoid negative outcomes such as onset of complications, hospitalizations, readmissions and mortality. Patient education not only allows diabetes individuals to gain knowledge on improving their health behavior such as changing unhealthy dietary habits, performing regular exercise, smoking or drinking cessation but also enhances medication or treatment compliance. Furthermore, previous studies showed that educating patients about self-management resulted in their loss of confidence about their health; thus, the goal of patient education was directed toward improving medication compliance [20,21]. Therefore, appropriate education or instruction to raise patients’ ability to manage their disease is essential. In particular, to allow patients to understand the concept of self-management, provision of early education is important. According to the American Diabetes Association, this education should be provided from the moment the patient is diagnosed with diabetes [14]. Thus, it seemed that the MCD incentive received within 1 year from diabetes diagnosis showed a significant association with hospitalization due to diabetes.

The results of the subgroup analyses showed that the low-income group were less likely to be associated with hospitalization due to diabetes as they received the MCD 10–12 times within the first year from the onset of diabetes. Additionally, the characteristics of the low-income group possibly contributed to this result. Previous studies have suggested that individuals in the low-income group usually have a lesser tendency to develop a healthy behavior than those in the high-income group [22]. Low socioeconomic status is known to be associated with poor health outcomes due to limited access to health care, under-utilization of preventive care and poor metabolic control [23]. Furthermore, low-income groups are known for having weak social support networks, which are important to disease management [24]; therefore, they cannot obtain affluent information about their chronic disease compared with the high-income group [25]. Thus, MCD could be used as a method to help patients understand their chronic disease and gain knowledge on how to manage their disease. Therefore, it seems that MCD received group showed relatively significant association with hospitalization due to diabetes compared with other income groups.

Besides income group, patients with diabetic complication showed lower HR for hospitalization due to diabetes when they received MCD. The number of MCDs received increased and the HR for hospitalization due to diabetes decreased; particularly, patients who received MCD 10–12 times per annum showed the lowest HR for hospitalization due to diabetes compared to patients in the MCD non-received group. Diabetes complication is associated with increased risk of hospitalization among diabetes patients [26]. Blood glucose control is also important to prevent patients with complication from aggravation [27]. Additionally, healthy behaviors are also associated with alleviate diabetic complications [28]. Therefore, MCD could play a role of enhancing patients to have better knowledge about blood sugar control and acknowledge the importance of healthy lifestyle. Based on these reasons, patients with complications were more likely to experience hospitalization due to diabetes if they do not receive the MCD.

The result of this study should be carefully interpreted as it has several limitations. First, we could not control the severity of diabetes. Hence, the diabetic complication index was adjusted. Second, we were not able to extract the data about the “fraud claims” from the patients’ medical records. Thus, “false claims” probably occurred, which means that physicians did not educate patients but wrote down on medical chart, for pretending they gave self-disease management education. Third, although we had washed out the start year of the sample cohort, we cannot stipulate that we only included those patients who were newly diagnosed with diabetes. Fourth, due to data limitations, we could not adjust other possible covariates such as educational level, marital status, living arrangement, occupation, or especially lifestyle behaviors such as diet or exercise which can influence the progression of diabetes and contribute to hospitalization. Fifth, we could not identify the contents of education the patients received from the physicians because the MCD was only defined by codes (AH200). Finally, the diabetes patients were selected using administrative claims data, which could contain coding errors, incomplete data and lack of clinical precision due to the characteristics of claims data.

## 5. Conclusions

According to the present study, MCD showed association with hospitalization due to diabetes. As the number of MCDs received increased, the HR for hospitalization due to diabetes decreased. Although some arguments demonstrate that the MCD incentive program is not useful as fraudulent claims have been brought up, self-management skills must be enhanced in order to control the progression of diabetes; thus, the results of this study are meaningful and may allow investigation of the outcomes of MCD. In future studies, the MCD incentive program must be well organized as it plays an important role in the improvement of patients’ self-managing skills.

## Figures and Tables

**Table 1:** General characteristics of the study population.

Variables	Total		Hospitalization Due to Diabetes				
			No		Yes		*p*-Value
	N	(%)	N	(%)	N	(%)	
**Management of Chronic Disease**							<0.0001
Non-received	46,571	86.2	40,368	86.7	6203	13.3	
1–3 times per annum	3694	6.8	3317	89.8	377	10.2	
4–6 times per annum	1284	2.4	1160	90.3	124	9.7	
7–9 times per annum	1164	2.2	1065	91.5	99	8.5	
10–12 times per annum	1318	2.4	1230	93.3	88	6.7	
**Management of Chronic Disease**							<0.0001
Non-received	46,571	86.2	40,368	86.7	6203	13.3	
Received	7460	13.8	6772	90.8	688	9.2	
**Gender**							<0.0001
Male	29,296	54.2	25,398	86.7	3898	13.3	
Female	24,735	45.8	21,742	87.9	2993	12.1	
**Income**							<0.0001
High	20,931	38.7	18,489	88.3	2442	11.7	
Middle	22,688	42.0	19,926	87.8	2762	12.2	
Low	10,412	19.3	8725	83.8	1687	16.2	
**Age group**							<0.0001
less than 50	21,781	40.3	20,049	92.1	1732	8.0	
50 to 59	14,533	26.9	12,798	88.1	1735	11.9	
60 to 69	10,881	20.1	8962	82.4	1919	17.6	
70 or over	6836	12.7	5331	78.0	1505	22.0	
**Existence of disorder**							<0.0001
No	50,018	92.6	43,886	87.7	6132	12.3	
Yes	4013	7.4	3254	81.1	759	18.9	
**Residential area**							<0.0001
Capital area	23,744	44.0	21,233	89.4	2511	10.6	
Metropolitan area	13,550	25.1	11,827	87.3	1723	12.7	
Rural area	16,737	31.0	14,080	84.1	2657	15.9	
**Type of insurance**							0.0005
Supporter	28,295	52.4	24,822	87.7	3473	12.3	
Dependent	25,736	47.6	22,318	86.7	3418	13.3	
**Existence of complication**							<0.0001
No	37,617	69.6	33,684	89.5	3933	10.5	
Yes	16,414	30.4	13,456	82.0	2958	18.0	
**Charlson Comorbidity Index**							<0.0001
None	9434	17.5	8641	91.6	793	8.4	
One	9707	18.0	8654	89.2	1053	10.9	
Two	9235	17.1	8146	88.2	1089	11.8	
Three or more	25,655	47.5	21,699	84.6	3956	15.4	
**Diabetes onset year**							<0.0001
2003	8643	16.0	6668	77.2	1975	22.9	
2004	6971	12.9	5720	82.1	1251	18.0	
2005	7011	13.0	5957	85.0	1054	15.0	
2006	5235	9.7	4591	87.7	644	12.3	
2007	4933	9.1	4432	89.8	501	10.2	
2008	5147	9.5	4458	86.6	689	13.4	
2009	4380	8.1	4000	91.3	380	8.7	
2010	3690	6.8	3502	94.9	188	5.1	
2011	4270	7.9	4103	96.1	167	3.9	
2012	3751	6.9	3709	98.9	42	1.1	
**Total**	54,031	100.0	47,140	87.3	6891	12.8	

**Table 2:** Factors associated with hospitalization due to diabetes by cox proportional hazards regression model.

Variables	Hospitalization Due to Diabetes		
	HR	95% CI	*p*-Value
**Management of Chronic Disease**			
Non-received	1.00	-	
1–3 times per annum	0.81	(0.73–0.90)	0.0001
4–6 times per annum	0.82	(0.68–0.98)	0.0248
7–9 times per annum	0.75	(0.62–0.92)	0.0054
10–12 times per annum	0.61	(0.50–0.76)	<0.0001
**Gender**			
Male	1.00	-	
Female	0.70	(0.67–0.74)	<.0001
**Income**			
Low	1.51	(1.41–1.61)	<0.0001
Middle	1.13	(1.07–1.19)	<0.0001
High	1.00	-	
**Age group**			
less than 50	1.00	-	
50 to 59	1.48	(1.38–1.58)	<0.0001
60 to 69	2.03	(1.90–2.17)	<0.0001
70 or over	3.29	(3.06–3.54)	<0.0001
**Existence of disorder**			
No	1.00	-	
Yes	1.46	(1.35–1.58)	<0.0001
**Residential area**			
Capital area	1.00	-	
Metropolitan area	1.22	(1.14–1.29)	<0.0001
Rural area	1.41	(1.34–1.49)	<0.0001
**Type of insurance**			
Supporter	1.00	-	
Dependent	1.04	(0.99–1.10)	0.1150
**Existence of complication**			
Yes	1.67	(1.59–1.75)	<0.0001
No	1.00	-	
**Charlson Comorbidity Index**			
None	1.00	-	
One	1.14	(1.04–1.25)	0.0051
Two	1.19	(1.09–1.31)	0.0002
Three or more	1.36	(1.26–1.47)	<0.0001
**Diabetes onset year**			
2003	1.17	(0.86–1.61)	0.3155
2004	1.06	(0.77–1.44)	0.7396
2005	1.02	(0.74–1.39)	0.9261
2006	0.96	(0.70–1.32)	0.8097
2007	0.91	(0.66–1.26)	0.5765
2008	1.30	(0.95–1.79)	0.1021
2009	1.25	(0.91–1.73)	0.1736
2010	1.07	(0.76–1.50)	0.6896
2011	1.14	(0.81–1.60)	0.4665
2012	1.00	-	

**Table 3:** Subgroup analyses of the association between management of chronic disease and hospitalization due to diabetes stratified by income, gender, age group, residential area and presence of complications.

Variables	Hospitalization Due to Diabetes								
	MCD Received	1–3 Times Per Accum		4–6 Times Per Accum		7–9 Times Per Accum		10–12 Times Per Accum	
	HR	HR	*p*-Value	HR	*p*-Value	HR	*p*-Value	HR	*p*-Value
Income *									
High	1.00	0.82	0.0354	0.72	0.0524	0.86	0.3656	0.69	0.0399
Middle	1.00	0.74	0.0007	0.92	0.5025	0.69	0.0165	0.68	0.0179
Low	1.00	0.86	0.1188	0.77	0.1883	0.79	0.2827	0.46	0.0010
Gender *									
Male	1.00	0.75	<0.0001	0.84	0.1317	0.66	0.0021	0.61	0.0002
Female	1.00	0.89	0.1300	0.76	0.0638	0.92	0.6031	0.61	0.0056
Age group *									
less than 50	1.00	0.94	0.5026	0.94	0.7243	0.89	0.5145	0.45	0.0009
50 to 59	1.00	0.74	0.0059	1.00	0.9853	0.79	0.1790	0.58	0.0073
60 to 69	1.00	0.58	<0.0001	0.61	0.0115	0.56	0.0119	0.78	0.1615
70 or over	1.00	1.05	0.6613	0.60	0.0323	0.78	0.3326	0.61	0.0792
Residential area *									
Capital area	1.00	0.78	0.0041	0.71	0.0283	0.86	0.3341	0.58	0.0029
Metropolitan area	1.00	0.86	0.1316	0.88	0.4911	0.76	0.1579	0.63	0.0187
Rural area	1.00	0.83	0.0327	0.88	0.3827	0.63	0.0155	0.64	0.0136
Existence of complication *									
Yes	1.00	0.68	<0.0001	0.57	0.0008	0.48	0.0001	0.40	<0.0001
No	1.00	0.91	0.1325	0.98	0.8618	0.96	0.7515	0.76	0.0317

* All variables in Table 1 was adjusted for analyses except the variables used for stratification.

## Supplementary Materials

The following are available online at http://www.mdpi.com/1660-4601/15/11/2541/s1.
