# Supplementary material for: Management of Chronic Disease and Hospitalization Due to Diabetes among Type 2 Diabetes Patients in Korea: Using the National Sample Cohort Data 2002–2013
Source: Int J Environ Res Public Health. 2018 Nov 13;15(11):2541. doi: 10.3390/ijerph15112541 (PMC6266696; doi:10.3390/ijerph15112541)
Supplement: Supplementary file 1 [file ijerph-15-02541-s001.pdf]

Supplementary Table S1. The general characteristics of the study population by Management of Chronic Disease recipient.

Variables	Total		Management of Chronic Disease				p-value
			No		Yes		
	N	(%)	N	(%)	N	(%)	
**All-cause hospitalization**							<.0001
No	28,167	52.1	23,552	83.6	4,615	16.4	
Yes	25,864	47.9	23,019	89.0	2,845	11.0	
**Hospitalization due to diabetes**							<.0001
No	47,140	87.3	40,368	85.6	6,772	14.4	
Yes	6,891	12.8	6,203	90.0	688	10.0	
**Gender**							<.0001
Male	29,296	54.2	24,911	85.0	4,385	15.0	
Female	24,735	45.8	21,660	87.6	3,075	12.4	
**Income**							<.0001
High	20,931	38.7	18,210	87.0	2,721	13.0	
Middle	22,688	42.0	19,473	85.8	3,215	14.2	
Low	10,412	19.3	8,888	85.4	1,524	14.6	
**Age group**							<.0001
less than 50	21,781	40.3	18,816	86.4	2,965	13.6	
50 to 59	14,533	26.9	12,326	84.8	2,207	15.2	
60 to 69	10,881	20.1	9,410	86.5	1,471	13.5	
70 or over	6,836	12.7	6,019	88.1	817	12.0	
**Existence of disorder**							0.3771
No	50,018	92.6	43,093	86.2	6,925	13.9	
Yes	4,013	7.4	3,478	86.7	535	13.3	
**Residential area**							0.0006
Capital area	23,744	44.0	20,405	85.9	3,339	14.1	
Metropolitan area	13,550	25.1	11,601	85.6	1,949	14.4	
Rural area	16,737	31.0	14,565	87.0	2,172	13.0	
**Type of insurance**							<.0001
Supporter	28,295	52.4	24,028	84.9	4,267	15.1	
Dependent	25,736	47.6	22,543	87.6	3,193	12.4	
**Existence of complication**							0.008
No	37,617	69.6	32,325	85.9	5,292	14.1	
Yes	16,414	30.4	14,246	86.8	2,168	13.2	
**Charlson Comorbidity Index**							<.0001
None	9,434	17.5	7,837	83.1	1,597	16.9	
One	9,707	18.0	8,230	84.8	1,477	15.2	
Two	9,235	17.1	7,858	85.1	1,377	14.9	
Three or more	25,655	47.5	22,646	88.3	3,009	11.7	
**Diabetes onset year**							
2003	8,643	16.0	7,580	87.7	1,063	12.3	
2004	6,971	12.9	6,179	88.6	792	11.4	
2005	7,011	13.0	6,161	87.9	850	12.1	
2006	5,235	9.7	4,612	88.1	623	11.9	
2007	4,933	9.1	4,275	86.7	658	13.3	
2008	5,147	9.5	4,418	85.8	729	14.2	
2009	4,380	8.1	3,749	85.6	631	14.4	
2010	3,690	6.8	3,082	83.5	608	16.5	
2011	4,270	7.9	3,478	81.5	792	18.6	
2012	3,751	6.9	3,037	81.0	714	19.0	
**Total**	54,031	100.0	46,571	86.2	7,460	13.8	

Supplementary Table S2. The association between the Management of Chronic Disease and all-cause hospitalization.

Variables	Total (n=54,031)		All-cause hospitalization								
			No (n=28,167)		Yes (n=25,864)		p-value	HR	95% CI		p-value
	N	(%)	N	(%)	N	(%)					
**Management of Chronic Disease**							<.0001				
Non-received	46,571	86.2	23,552	50.6	23,019	49.4		1.00	-		
1-3 times per annum	3,694	6.8	2,156	58.4	1,538	41.6		0.87	(0.82 -	0.91)	<.0001
4-6 times per annum	1,284	2.4	777	60.5	507	39.5		0.85	(0.78 -	0.93)	0.0003
7-9 times per annum	1,164	2.2	761	65.4	403	34.6		0.74	(0.67 -	0.82)	<.0001
10-12 times per annum	1,318	2.4	921	69.9	397	30.1		0.66	(0.59 -	0.72)	<.0001
**Gender**							<.0001				
Male	29,296	54.2	16,055	54.8	13,241	45.2		1.00	-		
Female	24,735	45.8	12,112	49.0	12,623	51.0		1.00	(0.97 -	1.02)	0.7138
**Income**							<.0001				
High	20,931	38.7	11,083	53.0	9,848	47.1		1.15	(1.11 -	1.19)	<.0001
Middle	22,688	42.0	11,868	52.3	10,820	47.7		1.07	(1.04 -	1.10)	<.0001
Low	10,412	19.3	5,216	50.1	5,196	49.9		1.00	-		
**Age group**							<.0001				
less than 50	21,781	40.3	13,123	60.3	8,658	39.8		1.00	-		
50 to 59	14,533	26.9	7,832	53.9	6,701	46.1		1.18	(1.14 -	1.22)	<.0001
60 to 69	10,881	20.1	4,632	42.6	6,249	57.4		1.44	(1.39 -	1.49)	<.0001
70 or over	6,836	12.7	2,580	37.7	4,256	62.3		2.06	(1.99 -	2.15)	<.0001
**Existence of disorder**							<.0001				
No	50,018	92.6	26,382	52.8	23,636	47.3		1.00	-		
Yes	4,013	7.4	1,785	44.5	2,228	55.5		1.29	(1.24 -	1.35)	<.0001
**Residential area**							<.0001				
Capital area	23,744	44.0	13,080	55.1	10,664	44.9		1.00	-		
Metropolitan area	13,550	25.1	7,036	51.9	6,514	48.1		1.10	(1.07 -	1.13)	<.0001
Rural area	16,737	31.0	8,051	48.1	8,686	51.9		1.16	(1.13 -	1.19)	<.0001
**Type of insurance**							<.0001				
Supporter	28,295	52.4	15,515	54.8	12,780	45.2		1.00	-		
Dependent	25,736	47.6	12,652	49.2	13,084	50.8		1.02	(0.99 -	1.05)	0.1441

**Existence of complication**							<.0001			
No	37,617	69.6	20,404	54.2	17,213	45.8		1.16	(1.13 - 1.19)	<.0001
Yes	16,414	30.4	7,763	47.3	8,651	52.7		1.00	-	
**Charlson Comorbidity Index**							<.0001			
None	9,434	17.5	6,572	69.7	2,862	30.3		1.00	-	
One	9,707	18.0	5,795	59.7	3,912	40.3		1.29	(1.23 - 1.36)	<.0001
Two	9,235	17.1	5,159	55.9	4,076	44.1		1.42	(1.36 - 1.49)	<.0001
Three or more	25,655	47.5	10,641	41.5	15,014	58.5		1.90	(1.83 - 1.98)	<.0001
**Diabetes onset year**							<.0001			
2003	8,643	16.0	2,798	32.4	5,845	67.6		0.43	(0.38 - 0.48)	<.0001
2004	6,971	12.9	2,650	38.0	4,321	62.0		0.46	(0.41 - 0.52)	<.0001
2005	7,011	13.0	2,963	42.3	4,048	57.7		0.50	(0.45 - 0.57)	<.0001
2006	5,235	9.7	2,452	46.8	2,783	53.2		0.55	(0.49 - 0.62)	<.0001
2007	4,933	9.1	2,571	52.1	2,362	47.9		0.59	(0.52 - 0.66)	<.0001
2008	5,147	9.5	2,719	52.8	2,428	47.2		0.69	(0.61 - 0.78)	<.0001
2009	4,380	8.1	2,748	62.7	1,632	37.3		0.74	(0.66 - 0.84)	<.0001
2010	3,690	6.8	2,579	69.9	1,111	30.1		0.84	(0.74 - 0.96)	0.0076
2011	4,270	7.9	3,265	76.5	1,005	23.5		0.99	(0.87 - 1.12)	0.8083
2012	3,751	6.9	3,422	91.2	329	8.8		1.00	-	
